# Satisfaction of patients with directly observed treatment strategy in Addis Ababa, Ethiopia: A mixed-methods study

**DOI:** 10.1371/journal.pone.0171209

**Published:** 2017-02-09

**Authors:** Belete Getahun, Zethu Zerish Nkosi

**Affiliations:** 1 University of South Africa, College of Human Science, Department of Health studies, Pretoria, South Africa; 2 Addis Ababa University, College of Health Sciences, School of Public Health, Addis Ababa, Ethiopia; McGill University, CANADA

## Abstract

**Background:**

Directly observed treatment, short course (DOTS) strategy has been a cornerstone for Tuberculosis (TB) control programs in developing countries. However, in Ethiopia satisfaction level of patients’ with TB with the this strategy is not well understood. Therefore, the study aimed to assess the satisfaction level of patients with TB with the DOTS.

**Method:**

Explanatory sequential mixed method design was carried out in Addis Ababa, Ethiopia. Interviewer-administered questionnaire with 601 patients with TB who were on follow-up was employed in the quantitative approach. In the qualitative approach telephonic-interview with 25 persons lost to follow-up and focus group discussions with 23 TB experts were conducted.

**Result:**

Sixty seven percent of respondent was satisfied with the DOTS. Rural residency (AOR = 3.4, 95% CI 1.6, 7.6), having TB symptoms (AOR = 0.6, 95% CI 0.4, 0.94) and treatment supporter (AOR = 4.3, 95%CI 2.7, 6.8) were associated with satisfaction with DOTS.

In qualitative finding, all persons lost to follow-up were dissatisfied while TB experts enlightened lack of evidence to affirm the satisfaction level of patients with DOTS. Explored factors contributing to satisfaction include: on time availability of health care providers, DOTS service delivery process, general condition of health care facilities, nutritional support and transportation.

**Conclusion:**

DOTS is limited to satisfy patients with TB and lacks a consistent system that determines the satisfaction level of patients with TB. Therefore, DOTS strategy needs to have a system to captures patients’ satisfaction level to respond on areas that need progress to improve DOTS service quality.

## Background

One third of the world’s population, approximately 2 billion persons, is thought to be latently infected with Mycobacterium tuberculosis (M.*tb*); 9 million persons develop active disease attributable to M.*tb* infection annually. In 2015, there were an estimated 10.4 million incident cases of TB and 1.4 million deaths from Tuberculosis (TB) globally [1]. Globally, for the last two hundred years TB has killed more than any other infectious disease [2].

The burden of the TB is higher in 30 high TB burdened countries (HBCs) which account for 84% of all estimated cases worldwide. Among these India leads with the highest number of TB cases, and Ethiopia held the 11^th^ position in 2015 [1]. In the same year in Ethiopia World Health Organization (WHO) 2016 report, indicated that 137,960 TB cases were notified and 26 per 100,000 mortality due to TB was occurred [1].

In the mid-1990s, WHO developed and recommended Directly Observed Treatment Short-course (DOTS) strategy for TB treatment. It is an international recommended strategy that has been considered as a cornerstone for TB control programs in developing countries including Ethiopia [3,4]. In Ethiopia since 1992 the TB prevention and control program piloted and incorporated the DOTS strategy. The strategy has shown an impressive coverage throughout the country and reached 100% geographic and 95% health institutions level coverage in 2012 [5].

The DOTS startegy implementation in Ethiopia evaluated at different times based on the treatment outcome of patients with TB with out due attention to the patients’ satisfaction [6–12]. However, satisfaction measurement is a core component to design and evaluate in modern health care services, acknowledges the role of the patient as partners in health care services and decisions [13] and satisfying patients is one of the main health system outcomes [14]. The success of DOTS depends mainly on the quality of services provided and the satisfaction levels among patients with TB [15]. Since satisfied patients are more adherent to Health Care Providers’ (HCPs) recommendations and prescribed treatment [16]. Therefore, this study was conducted to assess the level of satisfaction of patients with TB in Addis Ababa, Ethiopia. To augment and prevent the skew-ness of satisfaction of patients with TB who were on follow-up, lost to follow-up patients were included and TB expert’s view also considered substantiating the satisfaction level of patients with TB.

## Materials and methods

The study was used explanatory sequential mixed method study design from September 2015 to November 2015 in Addis Ababa, Ethiopia.

### Study setting

Addis Ababa has a surface area of 540 Square kilometers. According to projection of Central Statistics Agency (CSA) of Ethiopia, in the year 2014 its population was about 3.2 million [17]. The population density is 5646 persons per Km^2^. Administratively, the Addis Ababa is divided into 10 sub-cities, which are in turn divided into 116 wereda, the smallest government administrative units [18].

Available health institutions in the city are 48 hospitals, of which 34 are private, 11 public and 3 non-governmental (NGO). Among 11 public hospitals, 6 are owned and managed by Addis Ababa City Administration Health Bureau (AACAHB), 3 by Federal Ministry Health of Ethiopia and 2 Police and Defense Ministers [19]. In addition, Addis Ababa contains 93 health centers which are managed by AACAHB and mainly focused on primary health care services, and 647 private clinics mainly engaged with clinical service for profit. The 2014 Annual Report of AACAHB indicated that among the available health institutions in the region 24 private, 5 non-governmental organizations (NGOs) and 92 governmental health institutions provide TB treatment by DOTS strategy [20].

### Quantitative: Population, sampling and sample size

One hundred twenty one health facilities which were providing DOTS services in Addis Ababa were categorised into three groups. The categories were formed based on the ownership of the health facilities such as government, NGO for not profit and private for profit health facilities. Among listed health facilities in each category, by rule of thumb, approximately 25% of health facilities were randomly selected. Totally 30 health facilities were selected, of which 23 were among government, 6 were among private for profit and 1 was among non-government for not profit categories.

The sample size was determined based single population proportion formula with the assumption of 50% expected satisfaction level of patients with TB, 0.05 error allowance, 1.96 two-sided critical value for 95% confidence interval level and 0.05 levels of satisfaction significance. Based on the above information the calculated sample size was 384. The calculated sample size, 384, multiplied by 1.5 for design effect compensation, and 5% contingency for non-response rate added. Hence the final sample size was 605. The determined sample size was proportionally allocated based on the number of patients with TB who were on follow-up at each health facility. Patients with TB who were on follow-up were randomly recruited while they came for follow-up at each selected health facilities until the allotted sample size was reached.

### Quantiative data collection and management

The quantitative data were captured using an interviewer-administered structured questionnaire. The questionnaire was prepared through depth review of literatures [14, 15,16, 21–26] and from Federal Ministry Health of Ethiopia TB and Leprosy control manual [27]. Donabedian’s quality assessment model in health care setting was used as a proxy framework to arrange measuring items of the questionnaire [28].

The questionnaire contains 23 measuring items in 5-point Likert scale. Of which 10 items were focused on the structure, 10 items were focused on the process and 3 items were focused on the outcome of the DOTS. In addition, the questionnaire contained one stand-alone overall satisfaction measuring scale item ranged from 0 to 10 scores. The item’s lowest score was zero and the maximum score was 10.

Initially the questionnaire was prepared in English and translated into Amharic language (local and official working language of Ethiopia) and was translated back to English. The questionnaire was piloted on 27 patients with TB who were on follow-up at a health facility which was not included in actual data collection sites. The questionnaire’s overall reliability test was done in the pilot and actual study. Cronbach’s alpha of 0.88 and 0.85 were obtained from the pilot and actual study, respectively. The structure, process, and outcome measuring items Cronbach alpha values were 0.78, 0.78 and 0.85 respectively.

**The data were collected from 601 consented patients with TB who were on follow-up of TB treatment. Data were collected by nurses who trained on the questionnaire and ethical aspect of human subject involved study. Data was double entered into Statistical Packages for Social Science version 21.0 for Windows (SPSS, Chicago, IL, USA).**

### Satisfaction level determination

The satisfaction level of the respondents was determined by two methods. The first method was by 23 measuring items included in Donabedian’s quality assessment in health care model. The mean score of 23 measuring items included in the questionnaire was calculated for each respondent. The calculated mean score equal 2 and less were classed as unsatisfied and the mean score 4 and above were classed as satisfied. For scores between greater than 2 and less than 4, first the median was calculated then the scores less than the median were classed as unsatisfied and the rest classed as satisfied [21].

The second method was by taking the score of stand-alone single overall satisfaction measuring item. A stand-alone single overall satisfaction measuring scale mean was calculated and the mean score was used as cut of value to regard satisfied against dissatisfied [22]. However, the single stand-alone overall measuring item and 23 measuring item satisfaction level of the patients showed a significant difference (r = 0.41, p = 0.01) hence the researcher chooses Donabedian quality health care proxy framework calculated satisfaction level for further analysis.

The cut of value calculated from Donabedian quality health care proxy model was used to categorize satisfied against not satisfied for bivariate and multi-variable analysis. Predictors for patient satisfaction extracted from the literature related to DOTS strategy were assessed in binary logistic regression. In multi-variable regression variable which had a p value 0.05 in binary logistic regression were considered.

In order to keep instrument validation and reliability, a questionnaire was piloted before the actual data collection and Cronbach’s value was calculated. To increase the quality of data, (a) the data collectors were nurses who have proximity with TB treatment strategy, (b) a one day training was given for data collectors before the start of data collection, (c) the overall activities of data collection were monitored by the researcher and there was strict supervision during data collection, (d) all completed questionnaires were examined by the researcher for completeness (e) double data entryentry was done and consistencies of the collected data were checked during analysis.

### Qualitative: Population and sampling

In the qualitative approach, two groups of participants were included: persons lost to follow-up and TB experts. Before data collection, the TB register of each selected health facilities was checked to identify persons lost to follow-up within the six months’ time and their telephone address. A total of 23 TB experts was purposely selected. Two TB experts from each 10 Subcity health offices and 3 TB experts from AACAHB were included.

### Qualitative data collection and management

Focus group discussions (FGDs) and telephonic interview guide were developed in English based on literature review for TB experts and persons lost to follow-up, respectively. The FGD and telephonic interview guides, then translated to Amharic. Both the FGDs and telephonic interviews were facilitated in Amharic language. The qualitative data saturation was reached with 25 persons lost to follow-up patients with TB and 23 TB experts' participation during the interview and discussions, respectively [29].

Three FGDs with consented TB experts were conducted. The first and the second FGDs included 8 participants each while the third FGD included 7 participants. The discussions were facilitated based on pre-prepared leading questions. The leading questions were: how TB treatment is being provided in Addis Ababa, what are the challenges with DOTS strategy, how does/did you know either patients with TB are being satisfied or not, what are the factors that may contribute or limit satisfaction of patients with TB and what does/did you suggest to improve satisfaction levels of patient with TB. The FGDs were recorded and notes were taken.

Telephonic-interviews with consented persons lost to follow-up from TB treatments were carried out. In the interview probing questions were used. The probing questions were: what extent were you satisfied with the service provided while you were on follow-up of your treatment; what are the factors that made you satisfied/dissatisfied you, and what do you suggest to improve satisfaction of patient with TB.

### Qualitative data analysis

Qualitative data analysis was started simultaneously with the data collection. During data collection, field notes were taken, audio digital recorder (ADR) and non-verbal and gestural cues were observed. All Amharic recorded data were translated and transcribed verbatim by the researcher and research assistant independently. After the verbatim transcription and translation done, the data were compiled, read and re-read of the transcripts and emerging ideas were listed. Then codes, categories and sub-categories for the listed ideas were created. The categories were related by drawing lines between categories. Recoding was done when necessary. Themes were generated from these categories. The transcripts were also given to a public health specialist, who was also an expert in qualitative research methodology to independently create codes and categories, and identify emerging themes. A consensus meeting between the researcher and the public health specialist was held, where the categories and themes identified were compared, revised and then used as research findings for this study [30].

### Credibility, reliability and trustworthiness

The credibility, reliability and transferability of the study findings were enhanced with a collection of data from multiple sources: patients with TB who were on follow-up, lost to follow-up and TB experts. The study used different methods, quantitative and qualitative approaches using structured interview, telephonic-interview and FGD, and the findings were triangulated during interpretation. Study sites exhibited a varied number of patients with TB and only few numbers of respondents’ responses were collected per day for three months to reduce single day’s episodic feeling and recruit adequate number of participants.

The respondents were informed that they would not face anything that hinders her/his treatment at the time of data collection, during their treatment and beyond due to participating in the interview. So that enables the respondents to describe their real feeling. Moreover, verbatim translation of FGDs was shared with the participants to increase acceptability and dependability of the discussions.

This research extracted information employing questionnaires to patients with TB who were on follow-up, telephonic-interview of persons lost-to follow-up and FGD TB experts. After each sort of data set separately analysed, the findings were triangulated at an interpretation level to complement the weakness of one source of data over the other [30].

### Ethical consideration

Ethical clearance from Institutional Research and Higher Degrees committee from the University of South Africa, and permission letter from Addis Ababa City Adminstration Health Buraeu were obtained before the time of data collection. The IRBs at both institutions reviewed and apporved the consent procedures. Data were collected after securing informed written consent from every participant. The confidentiality of information was maintained by excluding personal identifiers, interviewing privately, and the signed consent forms are stored in lockable board at researcher office.

### Operational definition

Patient satisfaction: patients’ emotions, feelings and their perception of delivered DOTS services.

Directly observed treatment (DOT): means a health worker play an active role to ensure that every patient takes the recommended drugs, in the right combinations, on the correct schedule, for the appropriate duration [27].

## Result

Our result presented as follows: quantitative study findings: general characteristics of patients with TB, type of TB and the satisfaction level of the patient with TB and logistic regression analysis outcome. Then followed by qualitative study findings.

### Quantitative study findings

#### General characteristics of respondents

A total of 601 patients with TB were responded to the interviewer-administer a questionnaire, which accounts for 99.3% of the total expected respondents. Of these, 398 (66%) respondents were on intensive phase and 203 (34%) were in the continuation phase of the TB treatment. Three hundred thirty six (56%) of respondents were male (Table 1).

**Table 1:** Socio demographic characteristics of the respondents (N = 601).

Items		Frequency	Percentage	Cumulative percentage
Gender	Male	336	56	56
	Female	265	44	100
Age in years	18–24	127	21.1	21.1
	25–34	219	36.4	57.5
	35–44	148	24.6	82.1
	45–54	58	9.7	91.8
	55–64	31	5.2	97
	>65	18	3.0	100
Marital status	Married	255	42.4	42.4
	Single (Never married)	278	46.3	88.7
	Separated	12	2.0	90.7
	Divorced	20	3.3	94.0
	Widowed	28	4.7	98.7
	Cohabiting	8	1.3	100.0
Educational level	Diploma and above	143	23.8	23.8
	Secondary school(9–12)	196	32.6	56.4
	Primary school (5–8)	145	24.1	80.5
	Primary school (1–4)	47	7.8	88.4
	No formal education	70	11.6	100.0
Religious affiliation	Orthodox	401	66.8	66.8
	Muslim	117	19.3	86.1
	Protestant	74	12.4	98.5
	Catholic	8	1.3	99.8
	No religion	1	0.2	100.0

The respondents type of TB were 263 (43.8%) smear-positive pulmonary TB (PTB), 147 (24.4%) smear negative PTB, 159 (26.5%) Extra pulmonary TB (EPTB), and 32 (5.3%) multi drug resistance (MDR) TB. Regarding treatment category, 501 (83.4%) were new, 68 (11.3%) were relapse, 12 (2%) were after treatment failure, 4 (0.7%) were returned after lost to follow-up, and 13 (2.2%) were transferred respondents.

#### Satisfaction level of the respondents

**Fig 1:**
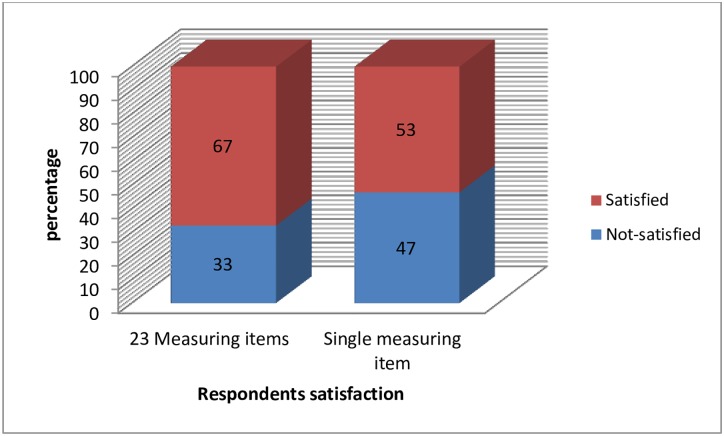
Respondents’ satisfaction level using 23 measuring items and overall single measuring item.

Sixty seven percent of the respondents were satisfied with the DOTS using the Donabedian quality health care model framed measuring items while 53% of the respondents were satisfied using the overall single measuring item (Fig 1). The respondents' satisfaction level based on the overall single measuring item and Donabedian quality health care model based measuring items score shows positive correlation (r = 0.419). However, they yield significantly different satisfaction level of the respondents (p = 0.05).

Among the 23 satisfaction measuring items, reduction of TB symptoms mean score was the highest of all measuring items, 4.16 (Standard Deviation, SD = 0.85) and the lowest mean score of measuring items was friendly-ness of the TB treatment provision environment, 3.13 (SD = 1.3) (Table 2).

**Table 2:** Mean scores of satisfaction measuring items.

Measuring items	Very dissatisfied *f (%)*	Dissatisfied*f (%)*	Neutral *f (%)*	Satisfied *f (%)*	Very Satisfied *f (%)*	Mean (SD)
**Structure; you are satisfied with:**						
treatment room privacy	11 (1.8)	23 (3.8)	44 (7.3)	387 (64.4)	136 (22.6)	4.02 (0.78)
access of the HCPs	15 (2.5)	44 (7.3)	32 (5.3)	365 (60.7)	145 (24.1)	3.96 (0.90)
safeness of the facility	20 (3.3)	36 (6)	37 (6.2)	364 (60.6)	144 (24)	3.95 (0.91)
easy access to re fill drugs	27 (4.5)	38 (6.3)	37 (6.2)	368 (61.2)	131 (21.8)	3.89 (0.96)
the waiting, registration and treatment room	30 (5)	49 (8.2)	52 (8.7)	357 (59.4)	112 (18.6)	3.78 (1.00)
availability of signage/ directions	49 (8.2)	47 (7.8)	36 (6)	337 (56.1)	132 (22)	3.75 (1.12)
availability of necessary drugs, laboratory reagents	31 (5.2)	51 (8.5)	87 (14.5)	312 (51.9)	120 (20)	3.73 (1.03)
cleanness, goodness and working order of the latrine	45 (7.5)	65 (10.8)	87 (14.5)	289 (48.1)	115 (19.1)	3.60 (1.13)
availability of safe water to ingest in pills	83 (13.8)	109 (18.1)	62 (10.3)	240 (39.9)	107 (17.8)	3.29 (1.32)
friendliness of the environment	100 (16.6)	107 (17.8)	75 (12.8)	252 (41.9)	67 (11.1)	3.13 (1.30)
**Process; you are satisfied with:**						
explanation and response of HCPs to questions	17 (2.8)	23 (3.8)	25 (4.2)	375 (62.4)	160 (26.6)	4.06 (0.85)
HCPs welcoming, respect and courteous	13 (2.2)	32 (5.3)	25 (4.2)	379 (63.1)	152 (25.3)	4.03 (0.84)
HCPs ability of the diagnosis, treatment and care of TB	14 (2.3)	32 (5.3)	43 (7.2)	357 (59.4)	155 (25.8)	4.0 (1.00)
appointment system	45 (7.5)	87 (14.5)	73 (12.1)	302 (50.2)	94 (15.6)	3.98 (0.83)
carefulness and allotted time of HCPs to check clinical condition	17 (2.8)	37 (6.2)	47 (7.8)	355 (49.1)	145 (24.1)	3.95 (0.90)
the cost you paid for TB diagnosis and treatment	34 (5.7)	47 (7.8)	55 (9.2)	316 (52.6)	149 (24.8)	3.83 (1.06)
HCPs usage of medical terms/jargon	51 (8.5)	71 (11.8)	38 (6.3)	314 (52.2)	127 (21.1)	3.65 (1.18)
HCPs considerer you are unwise	45 (7.5)	87 (14.5)	73 (12.1)	302 (50.2)	94 (15.6)	3.52 (1.14)
waiting time for processes	108 (18)	294 (48.9)	65 (10.8)	75 (12.5)	59 (9.8)	3.52 (1.20)
costs paid for transport	76 (12.6)	101 (16.8)	95 (15.8)	227 (37.8)	102 (17)	3.29 (1.28)
**Outcome; you are satisfied with your:**						
TB Symptoms reduction	12 (2)	24 (4)	30 (5)	322 (53.6)	213 (35.4)	4.16 (0.85)
psychological wellbeing-ness	11 (1.8)	36 (6)	20 (3.3)	330 (54.9)	204 (33.9)	4.13 (0.87)
physical wellbeing-ness	12 (2)	33 (5.5)	33 (5.5)	325 (54.1)	198 (32.9)	4.10 (0.87)
**Total mean score**						**3.80 (0.5)**

Among 601 respondents, 406 (67.7%) respondents were satisfied with DOTS. Of the 406 satisfied respondents, 208 (51.2%) were male respondents. There was a significant association between the satisfaction level and gender (p = 0.001). Among the 406 satisfied respondents, 170 (42%) respondents were with smear positive PTB, 108 (27%) were with smear negative PTB, 105 (26%) were with EPTB and 23 (5%) were with MDR-TB. There was no significant association between satisfaction with DOTS and type of TB (p = 0.28).

**Fig 2:**
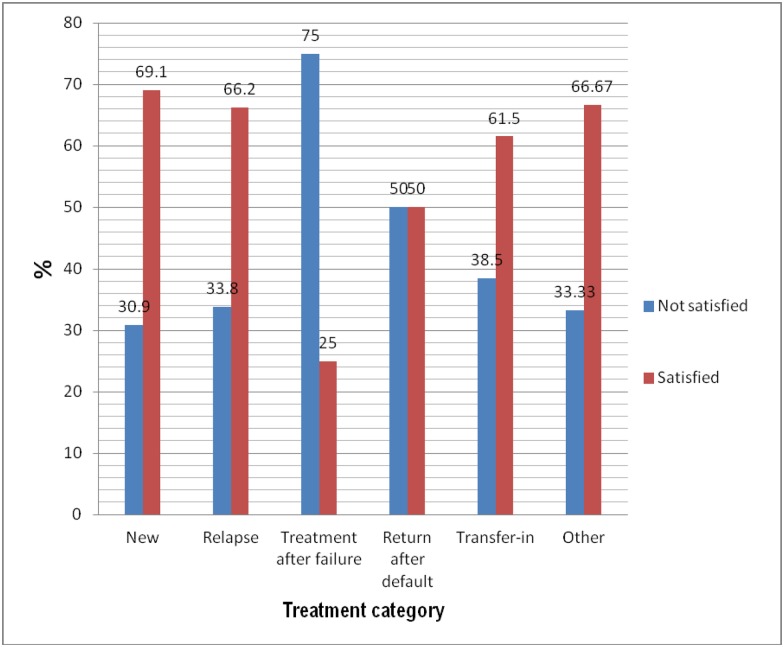
Level of satisfaction with treatment category of patients with TB (N = 601).

Among 195 dissatisfied respondents, 155 were new patient with TB, 23 were relapse patients with TB, 9 were treatment after failure patients with TB, 2 were returning after lost to follow-up patients with TB and 5 were transferred-in patients with TB (Fig 2). There was a marginally significant association among treatment categories and satisfaction of the patient with TB (p = 0.06).

Among 406 satisfied patients with TB, 93 (15.5%) were diploma and above holders, 129 (21.5%) were secondary school (9–12), 100 (16.6%) were second cycle primary school (5–8), 33 (5.5%) were first cycle primary school (1–4), and 51 (8.5%) had no formal education. There was no significant association between higher education level achieved and satisfaction (p> 0.05).

Age category of patient with TB was not significantly associated with satisfaction (p>0.05). Among 406 satisfied patients with TB, 82 (13.6%) were between 18–24 age category, 145 (24.1%) were between 25–34 age category, 106 (17.6%) were between 35–44 age category, 41 (6.8%) were 45–54 age category, 23 (3.8%) were 55–64 age category and 9 (1.5%) were above 65 years old.

There was a significant association between income category and satisfaction with DOTS (p = 0.02). Cross tabulation of satisfaction with income category presented in Table 3.

**Table 3:** Average family income of the respondents with satisfaction (N = 546).

		Average monthly income category in USD				Total
		9.4–47.0	47.1–95.0	95.01–143.0	143.1–957.0	
Not satisfied	Count	48	56	19	51	174
	% within Satisfaction level	27.6	32.2	10.9	29.3	100
	% within Average monthly income	26.4	38.1	23.5	37.5	31.9
	% of Total	8.8	10.3	3.5	9.3	31.9
Satisfied	Count	134	91	62	85	372
	% within Satisfaction level	36.0	24.5	16.7	22.8	100
	% within Average monthly income	73.6	61.9	76.5	62.5	68.1
	% of Total	24.5	16.7	11.4	15.6	68.1
TotalCount		182	147	81	136	546
% within Satisfaction level		33.3	26.9	14.8	24.9	100
% of Total		33.3	26.9	14.8	24.9	100

#### Regression analysis

Among 7 independent variables which showed an association with the satisfaction level in binary logistic regression, gender, presence of TB symptoms and treatment supporter were associated with satisfaction after controlling confounders (Table 4). The goodness of the model was assessed by Hosmer and Lemeshow Test, X^2^ = 5.904, df = 8 and p = 0.658 revealed the model adequacy during the analysis.

**Table 4:** Factors associated with satifaction with DOTS.

	Variables	Satisfaction		COR (95% CI)	P-value	AOR (95% CI)	P value
		Satisfied	Not satisfied				
Gender	Male	208	128	1.00		1.00	
	Female	198	67	1.81(1.2,2.6)	0.001	2.2(1.3, 3.57)	0.001
Residence	Urban	340	171	1.00		1.00	
	Rural	56	13	2.2 (0.9,5.2)	0.016	3.4(1.6, 7.6)	0.003
	Homeless*	10	11	4.7 (1.6, 13.5)	0.080	1.1(0.3, 3.7)	0.78
TB symptoms presence	Yes	113	75	0.6 (0.43, 0.9)	0.009	0.6(0.4, 0.94)	0.02
	No	293	120	1.00		1.00	
Good Communication	Yes	375	169	1.00		1.00	
	No	31	26	1.8(1.0,3.2)	0.02	1.6(0.8, 3.3)	0.01
Occupation	Permanent employee	38	36	1.00		1.00	
	Self-employee	166	53	1.6(0.9, 2.7)	0.051	1.5(0.87, 2.7)	0.13
	Temporary employee	47	39	0.6(0.33, 1.1)	0.133	0.48(0.2, 0.9)	0.03
	House wife	57	17	1.7(0.9, 3.5)	0.096	1.0(0.4, 2.4)	0.88
	Unemployed	48	35	0.7(0.4, 1.3)	0.291	0.56(0.23, 1.3)	0.20
	Pensioner	12	11	0.5(0.2, 0.4)	0.238	0.56(0.2, 1.6)	0.28
	Student	8	4	1.0(0.2, 3.7)	0.930	1.3(0.11, 15)	0.82
Income in USD	9.40–47.0	134	48	1	0.022	1.00	
	47.01–95.0	91	56	0.6(0.4, 0.9)	0.024	0.6(0.3, 1.1)	0.15
	95.01–143	62	19	1.1(0.6, 2.1)	0.616	1.3(0.6, 2.6)	0.47
	143.01–957	85	51	0.59(0.3, 0.9)	0.035	0.6(0.3, 1.1)	0.15
Treatment supporter	Family member	53	71	1.00		1.00	
	HCPs	349	122	3.82.5, 5.7)	000	4.3 (2.7, 6.8)	000

HCPs: Health care providers; 1.00: reference category; COR: Crude Odds Ratio; AOR: Adjusted Odds Ratio; CI: confidence interval:

* No formal living houses both in urban and rural

### Qualitative study findings

Among 23 TB experts participated in the FGDs, 15 (65%) were male. Thirty five percent of TB experts’ age category was 20–30 year, 13% was 31–40 year, 43.5% was 41–50 year and 8.6% was 51–60 year. The FGDs participants’ TB treatment and control related work experiences were ranged 2–15 years with the median of 5.5 years. Thirteen (55%) of FGDs participants had Master degree and 12 (45%) hold Bachelor degree.

Among 25 persons lost to follow-up contacted for the telephonic-interview, 3 (12%) of contacted addresses were wrong, 2 (8%) did not pick-up for repeated telephone calls 1 (4%) did not volunteer to participate in the telephonic-interview and 19 (76%) were fully participated. From 19 fully participated in a telephonic-interview, 11 (58%) were male. The mean age of the telephonic-interview participants was 39.4 years (SD = 9 years).

#### Satisfaction of persons lost to follow-up

Telephonic-interview participants explained that they were dissatisfied with DOTS service while they were on follow-up. The dissatisfaction of persons lost to follow-up and factors explored with satisfactions is explained in the following categories: DOTS delivery process, general condition of health facilities, aligned services and health care providers (HCPs) related (Table 5).

**Table 5:** Categories and sub-categories identified in telephonic-interview participants.

Categories	Subcategories
DOTS delivery process	Registration Treatment Referral Diagnosis compatibility Treatment supporter Daily observation at health facility
General condition of HCOs	Convenience the compound Availability of chairs Access of toilet
Aligned services	Nutrition Ambulance
HCPs	Availability Communication Reception Respect Information provision

#### DOTS delivery process

Most of the telephonic-interview respondents explained that the TB diagnosis and examination was started immediately on arrival in which registration was done, without much time desecrated. Immediate registration and the process start day were explained as:

“As I went they registered me and I was seen by doctors. With this perspective, I want to say thank you.”

However, the cause of illness was not detected in the same day; lasted for seven days and the time elapsed to have an apparent TB diagnosis was displeasing. One of the telephonic-interview respondents explained the dissatisfaction due to elapsed time as follows:

“I started my examination on August 5 2015; I did not receive the definite diagnosis until August 12, 2015.”

Another discontented issue described in DOTS service delivery process was an obligation to bring a treatment supporter. This was explained by the telephonic-interview respondent as:

“After I diagnosed with TB, I should brought somebody on the behalf of me. Should not I be responsible for me?”

Besides, some of the registered as persons lost to follow-up, received controversial diagnosis result; in one health facility received a result which concluded having TB while had not or other disease in another facility. The controversy results exposed the patients to unwanted cost. One of the telephonic-interview participants explained the controversy and its financial consequences as follows:

“In one health facility I was told that I had TB but after few days, in another facility they rejected the findings as I had TB. I did not go to third facility due to money shortage.”

The existence of the incompatible diagnosis result between health facilities was declared as a cause for dissatisfaction by the telephonic-interview respondents. Two respondents explained the incompatibility of the diagnosis as follows:

“I went to health centre where you find my address and had been told that as I had Extra Pulmonary TB and stared TB treatment. After a week I went to private clinic and have been informed as I had no TB.”

The telephonic-interview respondents explained that the daily follow-up of TB treatment exposed them for different direct and indirect costs. Furthermore, the difficulty of the daily observation was a cause to lost to follow-up. The telephonic-interview participants explained as follows:

“I am factory employee; I should be available at 6 am since the service reach at this time, otherwise I had to pay 12 Ethiopian birr per trip to reach to the factory where I work even I may reach at 10 am or so. However, they obliged me to go health facility daily to take the drug.”

“I and my wife were supposed to take him to health centre daily to follow-up. I got few days’ work permission and took him but after that my wife alone could not take him since he was bad-tempered. Now sadly he is mad and on the road.”

In addition, the DOTS delivery system was criticized as it is resistant to change treatment facility where to follow once started at a given heath facility due to the provision of little attention to give referral paper. One of the telephonic-interview participants explained the resistance to give a referral paper as:

“I told him that my husband should have to will move to another place for work with me and to write me a referral paper. He asked me where?… he responded that I need to give him exact name of the facility where I would follow but I knew only the area but not specific health centre’s name.”

#### General condition of the health facilities

The general conditions of the HCOs were not so much appreciated by telephonic-interview respondents. The respondents explained that though there were improvements in availing chairs around the TB room, no water to take the drugs and clean toilet, which enables you to use cosily near to the treatment room. The respondent explained the premise inconvenience as:

“I had to go to health facility after I emptied my colon at home since there was no convenient toilet.”

#### Allied services

The telephonic-interview respondents explained that allied health care services such availing transport, food, spiritual and social support are limited, however, the availability of these services is imperative. As reported in the interview, the existing system has no place alongside the screening of patients with TB for a diagnosis other than TB and HIV. The respondents explained weak integration of DOTS with nutritional support and no transport for patients with TB, even for the seriously ill. The respondent explained the less availability of aligned services as:

“I saw the ambulance which stood in the compound and I requested the HCP to tell the driver but the HCP replied as this is only for bleeding mother.”

“I had seen that some of patients with TB were receiving nutrition and asked to give me too but the response was as I had no MDR-TB.”

#### Health care providers (HCPs) related

Telephonic-interview respondents explained that HCPs who were engaged in the DOTS service delivery were available in working hours though they were not so punctual. With this perspective, the majority of the respondents explained as they had a good feeling about HCPs availability. However, few of telephonic-interview respondents did not agree fully about HCPs availability. One of the telephonic-interview respondents explained the disagreement as follows:

“While I was following my treatment HCPs were available usually after 8 am though they appointed us at 7:30 am.”

However, when the patients arrived earlier than HCPs at TB treatment room they tend to contempt the patients.

“If I reached earlier than the HCPs I usually sit aside of the TB room to prevent myself from being visible to HCPs when they come in.”

The HCPs reception on the first day of the treatment was mentioned by telephonic-interview respondents as it is with due respect, in a polite way and provide required information but the days go on they may throw the drugs through the window. One of the telephonic-interview respondents explained the mood swing with respect and information provision as follows:

“I could not tell how the HCP received me, respect and advise… when I was diagnosed as I had TB but it lasts on the same day.”

However, majority of telephonic-interview respondents explained that HCPs were not devoted to provide TB care service. The disagreement views of telephonic-interview respondents explained as:

“The HCP started writing while I was talking with, even was not willing to see my face and respond.”

Whereas another respondent explained that the HCPs’ compassionated care as:

“He, the HCP, was so kind while I was tired; even he was the one who hold up me to get into the car at the first week of the treatment.”

The telephonic-interview respondents explained that HCPs commitment and psychological support to patients with TB was not consistent among themselves and across health institution. The respondents put the level of commitment at two extremes. The extremes are range from holding up the weak patient to getting into the car to turning the face while the patient's request or raise an issue. These extremes were explained by the respondent as:

“I remember that, the HCP gave me my drugs in the car, almost 50 metres far from TB room.”

Nonetheless, another respondent explained limited commitment of HCPs as:

“The HCP was waiting me until I really combat on to TB room window let alone provision of the drug a place where I was.”

#### TB experts view on satisfaction of patients with TB

The FGDs participants explained that there is no consistent and regular system in the region that specifically capture the satisfaction level of patients with TB except some fragmented systems that is not fully dedicated exclusively for patients with TB. The FGD participants explained their perception in the following categories: satisfaction survey exclusively for patients with TB and integrated patient satisfaction (Table 6).

**Table 6:** Categories and sub-categories identified in FGDs.

Categories	Subcategories
Satisfaction survey for patient with TB	Availability of survey Exit interview with patients with TB Indicator Tool Homogeneity Good reception Good communication
Integrated satisfaction survey	Writing on recording book Identifying tag Every year Suggestion box utilisation Satisfaction not objectively measured

#### Presence of satisfaction survey for patients with TB

The FGDs participants explained that there is no regular, similar and standardise satisfaction survey of patients with TB as a strategy to understand the satisfaction level of patients’ with TB particularly with DOTS strategy. The FGDs participants described unavailability in satisfaction survey exclusively for patients with TB as follows:

“There is no satisfaction evaluation scheme for the TB treatment individually.”

However, at some facilities there are fragmented ways of understanding the satisfaction level of patients with TB. Opinion recording books and suggestion boxes are obtained in the health facilities to capture the perceptions of patients. Random interview of patients with TB during supportive supervision also helps to understand the level of satisfaction of patients’ with TB. The practice so far tried to understand the satisfaction level of patients’ with TB at some facilities was explained in the discussion as:

“We conduct supervision quarterly. We interview the patients in person on the treatment they have been given by the professionals in the TB room. We also have the public opinions gathering boxes in every department room. Those who can write drop their opinions there and other uses the chance during the interviews.”

The recording books and suggestion boxes were criticised for their lack of effectiveness to measure the level of satisfaction objectively since either, so much dissatisfied or so exited patients usually write their feelings. The weakness of the recording book as a tool for satisfaction survey was explained by FGD participant’s personal experiences as a service user. The FGD participant explained as:

“I remember once, I went to a clinic to have my eyes treated and I was very up set. I wrote on paper my temper but forgot to drop it in the box. These are the kinds of opinions we are talking about and they may not be bias free enough to evaluate clients’ satisfaction.”

**The FGDs participants agreed that there is a need for satisfaction measuring mechanism that apply to patients with TB. The discussant suggests that the indicator of satisfaction survey to patients with TB need to be scrutinised, a way of understanding their satisfaction level and the time interval to be yearly, biannually or quarterly at which the level of satisfaction should be measured.**

## Discussion

Although the Ethiopian Federal Ministry of Health (FMOH) has given due attention and priority to the quality of health care service since the first health sector development plan in 1997, a health care quality strategy has been developed recently. The Ethiopia health care quality strategy has included patient satisfaction as one of success indicators for the provision of quality service for the patients at HCO [31]. Even during Ethiopian hospital performance recognition ceremony in Addis Ababa, Ethiopia, in 2016, the Ethiopian FMOH minster stressed that “the next selection of hospital’s award will be mainly based on survey of client satisfaction. Client satisfaction will be the main indicator.” In addition Ethiopia’s 2015–2020 health sector development plans focus on quality health care delivery and equity in health [32].

The study reveals different levels of satisfaction with DOTS among different study participant groups. The majority (67%) of patients with TB that were on follow-up of their TB treatment was satisfied with DOTS similar to elsewhere in Ethiopia [23] and in other countries [33–35]. However, all persons lost to follow-up to TB treatment and 33% of patients with TB who were on follow-up were dissatisfied. Despite the dissatisfaction with DOTS, 33% of patients with TB were following their TB treatment. This may indicate that the extent DOTS demand persistence of the patient to adhere with the treatment. Similar to the study conducted elsewhere in Ethiopia [23], the study shows that patients with TB who lost endurance to resist the dissatisfactions caused by DOTS were lost from follow-up of TB treatment.

Regular measuring and knowing the satisfaction level of the patients emphasized as indicated in quality service provision [33, 35]. However, the current study indicates that the DOTS implementation in the study area is not tied with a regular mechanism which captures the satisfaction level of patient with TB; except the presence of few fragmented capturing tools such as opinion recording books and a suggestion box. The use of opinion recording books and suggestion boxes are criticized because they are usually used by few highly excited patients with TB. Hence, they lack to indicate the satisfaction level of patient with TB and inform the health programmer and HCPs.

TB experts participated in this study could not affirm whether the dissatisfaction of patients with TB with DOTS existed. As a result, the study highlighted the importance of the presence of regular and consistent mechanism to understand satisfaction level of patients with TB across health facilities.

Similar with another study [36], gender of the patients in the present study showed a significant association with satisfaction. Female patients with TB were 2.2 times more likely to be satisfied with the service they received as compared to the male counterpart. In contrary, another study reported that although female had more tendencies to be satisfied than male, gender’s association with satisfaction was not statistically significant [37].

Similar to the study conducted in Khyber Pakhtunkhwa, the study shows that attributes of patient satisfaction from the side of HCPs are communication skills, technical skills and responsiveness of HCPs to patient needs [36]. In addition, similar to the study conducted in Ethiopia [23], the study shows that satisfaction level of patients with TB are influenced by on time availability and good reception of HCP, provision of information and respect to the patients and waiting time. Alike Pefoyo and Wodchis [38] suggested, the study indicates that the patients’ satisfaction is related with access and convenience of the HCOs general conditions such as availability of comfortable chair to get health education and clean toilets set up/facility. Alike other studies [23,39], this study shows that unavailability of consistent nutrition support and absence of transport for patients with TB, especially for seriously ill patients with TB was the cause for dissatisfaction; even they are highlighted as causes to not attend the treatment.

Residence, presence of TB symptoms and treatment supporter revealed a significant association with satisfaction with DOTS. Unlike to another study [40], in this study educational level are not associated with satisfaction level of patients with TB. However, highly educated patients are less likely to be satisfied than who has a lesser educational level due to expectation difference, patients with high expectations tend to be less satisfied with health care service [35].

The study included different study groups and described the level of patients with TB satisfaction from a different perspective by using quantitative and qualitative approaches. The outcome of the study can be generalized to different regions and countries at the primary health care level with similar settings. However, Addis Ababa is among the eleven geographic regions in the Ethiopian government structure, and is an autonomous administrative region and the largest and capital city of Ethiopia and the population is totally considered as urban dwellers (17). Hence, the majority of the respondents were urban dwellers; therefore, its generalization might be compromised for rural settings. In addition, satisfaction ratings in this study do not replace TSRs or other treatment outcome indicators in DOTS.

## Conclusion

The study finding shows that patients with TB who were on follow-up of their treatment were following their treatment with dissatisfaction. All of the lost to follow-up patients with TB were dissatisfied with DOTS. Dissatisfaction is one of the causes to deviate from follow-up. DOTS delivery process, general condition of HCO, allied health care services such as nutritional support and transport services are explored factors with satisfaction. Therefore, the DOTS strategy should include a regular mechanism that informs programmer, policy maker and HCPs about patients with TB satisfaction with DOTS. Moreover, the HCPs, TB-experts, Health programmer need to act on the barriers of patients with TB’ dissatisfaction such as unavailability of nutritional support and transport support, inconvenience of HCOs conditions and long waiting time in health facilities.

## Supporting information

S1 Satisfaction measurement questionnaire and interview guides (PDF)

## Abbreviations

AACAHB: Addis Ababa City Administration Health Bureau
CSA: Central Statistics Agency
DOT: Directly Observed Treatment
DOTS: Directly Observed Treatment-Short course
EFMOH: Ethiopian Federal Ministry of Health
EPHI: Ethiopian Public Health Institute
HBC: High TB Burdened Country
HCO: Health Care Organization
HCP: Health care providers
IUATL: International Union against Tuberculosis and Lung Disease
MDR-TB: Multi Drug Resistance TB
M.tb: Mycobacterium Tuberculosis
RHBs: Regional Health Bureaus
TB: Tuberculosis
UNISA: University of South Africa
WHO: World Health Organization
