# Supplementary material for: Satisfaction of patients with directly observed treatment strategy in Addis Ababa, Ethiopia: A mixed-methods study
Source: PLoS One. 2017 Feb 9;12(2):e0171209. doi: 10.1371/journal.pone.0171209 (PMC5300143; doi:10.1371/journal.pone.0171209)
Supplement: S1 Satisfaction measurement questionnaire and interview guides — (PDF) [file pone.0171209.s001.pdf]

Annex D: English version of patients with TB questionnaire

Part I. Instruction

1. Before collecting any information, the letter of ethical clearance and cooperation letter should be presented to the head/representative of the facility and get permission.
2. Before conducting interview the below study information sheet must be read to sure the respondents acquire the required knowledge whether to participate in interview or not. In addition, get the participant consent.
3. For all responses, it should be recorded what the respondent exactly wanted to say.
4. All the close-ended questions should be chosen and circled from the given choices unless and otherwise the response is different from listed choices.
5. If the response is different from the listed choices in close-ended questions, the response should be put in other and must be specified.
6. For open-ended questions, the response should be written in the form that have provided in the bracket at the end of each questions.
7. If the respondent has general comment it should be written at the end of questions on provided space.

Questions

Se. no	Measurement Items	Response
	**General characteristics**	
**1.**	Gender of the respondent	1. Male 2. Female
**2.**	Age	1. 18 - 24<br>2. 25 -34<br>3. 35 – 44<br>4. 45 – 54<br>5. 55- 64<br>6. 65 and above
**3**	Marital status	1. Married<br>2. Single (Never married)<br>3. Separated<br>4. Divorced<br>5. Widowed<br>6. Cohabiting
**4**	Family size?	_______ (including the respondent)
**5**	Residence	1. Urban 2. Urban slum 3. Rural 4. Homeless 5. Other (specify)
**6**	Highest educational level	1. Diploma and above<br>2. Preparatory<br>3. Secondary school<br>4. Primary school<br>5. No formal education
**7**	Occupation	1. Permanent employee<br>2. Self employee<br>3. Temporary employee<br>4. Unemployed<br>5. Pensioner
**8**	Average household/family monthly income	1 ___________ 2. No response
**9**	Religion	1. Orthodox 2. Protestant 3. Catholic 4. Muslim 5. No religion 6. Other
**10**	Ethnic group	1. Oromo<br>2. Amhara<br>3. Tigre<br>4. Gurage<br>5. Other (specify)
**11**	Did you receive health care service in this facility before this illness?	1. Yes 2. No
**12**	If yes, how many days did you visit the health institution before you diagnosed as you have TB?	______________
**13**	Type of TB	1. Pulmonary positive TB<br>2. Pulmonary Negative TB<br>3. Extra Pulmonary TB<br>4. MDR-TB
**14**	Treatment category/registration group (please see treatment follow up card)	1. New<br>2. Relapse<br>3. Treatment after Failure<br>4. Return after Defaulted<br>5. Transfer in<br>6. Other(specify)
**15**	When did you start TB treatment?	(DD/MM/YYYY)
**16**	Do you have TB symptoms now?	1. Yes 2. No
**17**	Total planed treatment duration?	1. Six months 2. Eight months 3. Other (specify)__________
**18**	Currently how you are collecting your TB drugs	1. Daily 2. Weekly<br>3. Monthly 4. Other
**19**	How much you pay for transport per day in ETB? (if there are care giver include all costs)	______________

**20**	Did you expect to come and collect the drugs every day, week or months for this much time?	1. Yes 2. No
**21**	Do you think that you and HCPs have good communication?	1. Yes 2. No
**22**	Who is your treatment supporter?	1. HCP<br>2. Family member<br>3. Health extension worker<br>4. Friend<br>5. Volunteer<br>6. Other (specify)

	**Satisfaction with TB care received**<br>**5. Very satisfied 4. Satisfied 3. Neutral 2. Dissatisfied 1. Very dissatisfied**					
**1**	**Structure**					
	Are you satisfied with:					
**1.1**	availability of necessary equipment, drugs, laboratory reagents to treat TB disease	**1**	**2**	**3**	**4**	**5**
**1.2**	easy access of the HCPs as you need	**1**	**2**	**3**	**4**	**5**
**1.3**	easy access of HCPs to re fill your medication	**1**	**2**	**3**	**4**	**5**
**1.4**	wheelchair friendliness of the environment	**1**	**2**	**3**	**4**	**5**
**1.5**	the waiting area, registration and treatment room comfortableness and availability of seats	**1**	**2**	**3**	**4**	**5**
**1.6**	the safeness of the facility for the patients	**1**	**2**	**3**	**4**	**5**
**1.7**	availability of signage/ directions guidance where to go in the health facility	**1**	**2**	**3**	**4**	**5**
**1.8**	cleanness, goodness and working order of the latrine	**1**	**2**	**3**	**4**	**5**
**1.9**	treatment room keeping your privacy	**1**	**2**	**3**	**4**	**5**
**1.10**	availability of safe water to take a medication	**1**	**2**	**3**	**4**	**5**
**2**	**Process**					
	Are you satisfied with:					
**2.1**	explanation and response of HCPs about your questions	**1**	**2**	**3**	**4**	**5**
**2.2**	HCPs ability of the diagnosis, treatment and care of TB	**1**	**2**	**3**	**4**	**5**
**2.3**	the cost you paid for TB diagnosis and treatment	**1**	**2**	**3**	**4**	**5**
**2.4**	obligation of costs beyond my ability to pay for transport	**1**	**2**	**3**	**4**	**5**
**2.5**	Carefulness and allotted time of HCPs to check my clinical condition	**1**	**2**	**3**	**4**	**5**
**2.6**	HCP welcoming, respect, friendly treatment and courteous	**1**	**2**	**3**	**4**	**5**
**2.7**	HCPs considerer you are unwise	**1**	**2**	**3**	**4**	**5**
**2.8**	appointment system for follow up	**1**	**2**	**3**	**4**	**5**
**2.9**	so long waiting time with registration process to get TB care	**1**	**2**	**3**	**4**	**5**
**2.10**	HCP uses medical terms/jurgon without explaining what they mean	**1**	**2**	**3**	**4**	**5**
**3**	**Out come**					
	Are you satisfied with:					
**3.1**	Your TB symptoms reduction rate	**1**	**2**	**3**	**4**	**5**
**3.2**	Physical wellbeing ness you gained due to TB treatment	**1**	**2**	**3**	**4**	**5**
**3.1**	Psychological wellbeing ness you gained due to TB treatment	**1**	**2**	**3**	**4**	**5**
**4.**	**General satisfaction**					
	How much you are satisfied by the care you received (the least is 0 and the highest is 10)	**0______________10**				

Thank you for your time and cooperation! Is there anything you would like to ask or say?

Data collector name and sign ________________________ Date _________________

Annex E: lost to follow-up patient with TB telephone interview guide

Hello.

Greetings, my name is Belete Getahun Woldeyes, have been working research on Evaluation of DOTS strategy patient centeredness and satisfaction in Addis Ababa, Ethiopia. The aim of the study is to evaluate lost to follow-up patientsTB satisfaction level in order to capture lessons that can be used in future interventions. In addition, the study will help to improve the programme helps to improve patient satisfaction

With the strateg.

Today I would like to talk to you and gather information through telephone about your experiences on DOTS programme in Addis Ababa as one of the components of this study. It takes 20 -25 minutes, though it seems long time your response will help the study greatly since you started and defaulted TBtreatment at ____________facility.

This study, ethically, has been approved by UNISAethical review board and get permission from Addis Ababa City Administration Health Bureau.

Your name will not be quoted and the responses will be kept confidential, only be shared for research team. You do not have to discuss issues that you do not want. If you want to withdraw from the study any time along the discussion process, you will not be obliged to continue or give reasons for doing so.

Do you have any question or which is not clear?

Do you participate in the study?

I would like to appreciate your help in responding to this interview. If you are comfortable let we start the discussion with:

1. How do you explain the overall structure, comfortableness of the health care facility where you diagnosed as you have TB and started treatment?
2. How was the relationship/link among one department with others in health care service organization: registration room, laboratory with treatment room?
3. What was/ were the service/s you used linked with TB treatment?
4. How you were assisted while you faced difficult situations in the TB care provision, if any?
5. How do you explain the TB care you received at health institution?
6. What was/were the reasons forced you to default from the TBtreatment?

7. At what extent you satisfied with the service provided to you while you were on following up your treatment; what are the factors?
8. What do you suggest to improve TB patients' satisfactions with service?
9. Is there anything more you would like to add?

Annex G: FGD interview guide

Guiding questions

1) How TB treatment is being provided in Addis Ababa for TB patients?
2) What are the challenges to DOTS strategy?
3) How TB patients select their treatment supporter?
4) How does/did you know either TB patients are being satisfied or not?
5) What are the factors that may contribute or limit TB patient satisfactions in intensive and continuation phase of the treatment?
6) What does/did you suggest to improve TB patients' satisfactions?
7) Is there anything more you would like to add?

I will be analySing the information you and others gave me and submitting a draft report to the Addis Ababa City Administration Health Bureau as we compiled it. I will be happy to send you a copy to review at that time, if you are interested.

Thank you for your time.

Annex H: Amharic version of the questionnaire for patients TB

ክፍል 1. መመሪያ

1. መረጃውን ማሰባሰብ ከመጀመሩ በፊት፣ ፈቃድ ለማግኘት የኤቲካል ክሊራንስ እንዲሁም የትብብር ደብዳቤ ለተቋም ሃላፊ ወይም ለተወካዩ መቅረብ ይኖርበታል፡፡

2. መጠየቁ ከመጀመሩ በፊት የመረጃው ወረቀት መነበብ ይኖርበታል፣ በተጨማሪም ተጠያቂው መጠየቁን ለመሳተፍ እንዲወስን በቂ እውቀት ማግኘቱን መረጋገጥ ይኖርበታል፡፡

3. የሁሉም ተጠያቂዎች መልስ ምን ማለት እንደሚፈልጉ ግልፅ በሆነ መልኩ መቀረፅ አለበት፡፡

4. ምርጫ ለተሰጣቸው ጥያቄዎች መልስዎን በማክበብ የጠቁሙ፣ መልስዎ ካልተጠቀሰ በስተቀር ለምርጫ ጥያቄዎች መልስዎ ከተጠቀሱት የተለየ ከሆነ የተለየ በሚለው እና መጠቀስ ይገባዋል

5. በፅሁፍ ለሚገለፁ ጥያቄዎች፣ መልስዎን በቅንፍ በተሰጡት የመልስ መስጫ ቦታዎች መልስዎን ይስጡ፡፡

6. አጠቃላይ አስተያየት ካለዎ የጥቄው መጨረሻ ላይ በተሰጠው ክፍት ቦታ ይፃፉ፡፡

መጠይቅ

ሀ. አጠቃላይ		
**ቁ.**	**ጥያቄ**	**መልስ**
1	ፆታ	1. ወንድ 2. ሴት
2	እድሜ በአመት	1. 18-24 2. 25-34 3. 35- 44 4. 45- 54 5. 55-64 6. 65 ና በላይ
3	የትዳር ሁኔታ	1. ያገባ 2. ያላገባ 3. የተለያየ 4. የፈታ 5. የሞተበት 6. አብሮ የሚኖሩ
4	የቤተሰብ ብዛትበቁጥር	
5	መኖሪያ	1.ከተማ 2. ገጠር 3. ቤት የሌለው
6	የትምህርት ደረጃ	1. ዲፕሎማ ና ከዚህ በላይ 2.ሁለተኛ ደረጃ (9-12) 3.የመጀመርያ (5-8) 4. የመጀመርያ(1-4) 5.መደበኛ ትምህርት ያልተማረ 6. የተለየ(ግለፅ)______________
7	ስራ	1. ቋሚ ሰራተኛ 2. የግል ስራ 3. ጊዜያዊ ቅጥር 4. የቤት እመቤት 5. ስራ አጥ 6. የተለየ(ግለፅ)________________
8	አማካኝ የቤተሰብ የወር ገቢ? (በብር)	
9	ሃይማኖት	1. ኦርቶዶክስ 2. ፕሮቴስታንት 3. ካቶሊክ 4. ሙስሊም 5. ሃይማኖት የሌለው 6. ሌላ(ግለፅ)____
10	ብሄር	1. ኦሮሞ 2. አማራ 3. ትግሬ 4.ጉራጌ 5. የተለየ(ግለፅ)_____________
11	ከዚህ በፊት እዚህ ታክመው ያውቃሉ?	1.አዎ 2. አይ
12	አዎ ከሆነ ለምን ያህል ቀን?	
13	የአለብዎ የቲቢ አይነት	1. በአክታ የተረጋገጠ የሳንባ ቲቢ 2. በአክታ ያልተረጋገጠ የሳንባ ቲቢ 3.ከሳንባ ዉጭ የሆነ ቲቢ 4. መደሃኒት የተላመደ ቲቢ
14	የበሽተኛው የህመም ክፍል የትኛው ነዉ?	1. አዲስ የቲቢ በሽተኛ 2. ቲቢ ያገረሸበት በሽተኛ 3. የቲቢ መድሃኒት ህክምና ያልሰራለት 4. የቲቢ ህክምና የቋረጠ 5. በሪፈራል የመጣ 6. ሌላ(ግለፅ)______
15	ህክምናመቼ ጀመሩ	(ቀን/ወር/ አመት)
16	አሁን የህመም ምልክት አለዎት?	1.አዎ 2. አይ
17	የታቀደው የህክምና ክትትል ጊዜ	1. ስድስት ወር 2. ስምንት ወር 3. የተለየ (ግለፅ)_____________.
18	አሁን ከጤና ድርጅት መድሃኒት የሚወስዱት?	1. በቀን በቀን 2. በሳምንት 3. በወር 4. የተለየ(ግለፅ)_____
19	ለዚህን ያህል ጊዜ በየቀኑ/በየሳምንቱ እመላለሳልሁ ብለው ገምተው ነበረ?	1.አዎ 2. አይ
20	በርሶና በህክምና ክትትል ሰጪዎ መካከል ጥሩ መግባባት አለ?	1.አዎ 2. አይ
21	የ ቲቢ ህክምና ረዳትዎ	1.የጤና ባለሙያ 2. የቤተሰብ አባል 3. የጤና እክስቴንሽን ሰራተኛ 4.ጓደኛ 5. በጎ ፈቃደኛ 6.የተለየ(ግለፅ)______

ሐ. የቲቢ ታካሚዎች እርካታ በቲቢ ህክምና አገልግሎት አሰጣጥ
5= በጣም ረክቻለሁ 4= ረክቻለሁ 3= ገለልተኛ 2= አልረካሁም 1= በጣም አልረካሁም

**1.**	**አወቃቀር:** በሚከተሉት እርካታ ይሰማዎታል?					
1.1	የቲቢ በሽታን ለማከም የሚያገለግሉ አስፈላጊ ቁሳቁስ፣ መድኃኒቶች፣ የላብራቶሪ ኬሚካሎች መገኘት	1	2	3	4	5
1.2	የክትትል ሰጪዎች በሚፈለጉበት ጊዜ መገኘት	1	2	3	4	5
1.3	መድሃኒትዎን መልሰ ለመሙላት የክትትል ሰጪዎች በቀላል መገኘት	1	2	3	4	5
1.4	አካባቢው ለዊልቸር ተስማሚ መሆን	1	2	3	4	5
1.5	የመጠበቂያ፣ የመመዝገቢያ እና የመታከሚያ ክፍሎች ምቹነትና የወንበሮች መገኘት	1	2	3	4	5
1.6	የሕክምና ቦታው ለታካሚዎች ያለው ደህነንት	1	2	3	4	5
1.7	የሕክምና ቦታው ውስጥ የት መሄድ እንዳለብዎት አቅጣጫ የሚያሳዩ መጠቁሞች መገኘት	1	2	3	4	5

1.8	የመጸዳጃቤቱ ንጽሕና፣ ጥሩነት	1	2	3	4	5
1.9	የሕክምና ክፍል ንፁህነት	1	2	3	4	5
1.10	ለመድሃኒት መዋጫ የሚሆን ደህንነቱ የተጠበቀ ውሀ መገኘት	1	2	3	4	5
**2**	**አሠራር**: በሚከተሉት እርካታ ይሰማዎታል?					
2.1	የእርስዎን ጥያቄዎች አስመልክቶ የጤና ክትትል ሰጪዎች የሚሰጡት ማብራሪያና ምላሽ	1	2	3	4	5
2.2	የጤና ክትትል ሰጪዎች የቲቢ ምርመራ፣ ሕክምና እና ክትትል ችሎታ	1	2	3	4	5
2.3	ለቲቢ ምርመራ እና ሕክምና የከፈሉት ገንዘብ ተመጣጣኝነት	1	2	3	4	5
2.4	ለመጓጓዣ ልከፍለው ከምችለው በላይ የመጡብኝ የክፍያ ግዴታዎች	1	2	3	4	5
2.5	የጤና ሁኔታዬ ደህንነት ለማረጋገጥ የጤና ክትትል ሰጪዎች የሚያደርጉት ጥንቃቄ እና የሚመድቡት ጊዜ	1	2	3	4	5
2.6	የጤና ክትትል ሰጪዎች አቀባል፣ አክብሮትና ተስማሚ እንክብካቤ	1	2	3	4	5
2.7	የጤና ክትትል ሰጪ ባለሙያዎች አንዳንደ እንደማያውቁ ይቆጥርዎታል	1	2	3	4	5
2.8	ለክትትልቀጠሮ ለመያዝ ያለው ስርዓት	1	2	3	4	5
2.9	የቲቢ ክትትል ለማግኘት ለመመዝገብ ረጅም ጊዜ መጠበቅ	1	2	3	4	5
2.10	የጤና ክትትል ሰጪው የሕክምና ቃላትን ሲጠቀም አያብራራም	1	2	3	4	5
**3**	**ዉጤት**: በሚከተሉት እርካታ ተሰምቶታል?					
3.1	በቲቢ ህክምና ባገኙ ት የህመም ስሜትዎ አቀናነስ	1	2	3	4	5
3.2	በቲቢ ህክምና ባገኙ ት አካላዊ ደህንነት	1	2	3	4	5
3.3	በቲቢህክምና ባገኙ ት የመንፈስ ደህንነት	1	2	3	4	5
**4.**	**አጠቃላይ እርካታ**: በተሰጠዎት ክትትል ምን ያክል ረኩ (ትንሹ 0 ትልቁ ደግሞ 10 ነው)	**0**________________10				

ግዜዎን ስለሰጡኝ አመሰግናለሁ ። ማለት የሚፈልጉት ካለ______________________________

የመረጃ ሰብሳቢው ስምና ፊርማ_____________ ቀን________________________________

<u>በኢትዮጵያ በቀጥታ የሚታይ የቲቢ ህክምና ስትራቴጂ ግምገማ፡ የበሽተኛን ማእከልነት እና የእርካታ ጥናት</u>

<u>የግሩፕ ውይይት መንደርደሪያ</u>

1) በአዲስ አበባ እየተሰጠ ያለው የቲቢ ህክምና ሂደት ምን ይመስላል?
2) በቀጥታ እየታየ የሚሰጠው የቲቢ ህክምና ተግዳሮቶቹ ምንድን ናቸው?
3) እንዴት ነው የቲቢ ተካሚዎች በቲቢ ህክምና አስጣጥ ይርኩ ወይ አይርኩ የሚታወቀው?
4) ምንድን ናቸው የተካሚዎን እርካታ የሚቀንሱ/እንዲጨምር የሚያደርጉት? ?
5) የታካሚውን እርካታ ለመጨመር ምን ቢደረግ ይሻላል?
6) ዋናው የቲቢ ተካሚዎችን ህክምና እንዲያቋርጡ የሚያደርጋቸው ነገር?
7) መጨመር ወይም ማለት የምትፈልጉት ካለ?

ግዜያችሁን ሰጥታችሁ ስለ ተሳተፋችህ አመሰግናለው:: እናንተና ሌሎች የሰጡኝን አጠናቅሬ ለጤና ቢሮ አቀርባለው፡፡

አመሰግናለሁ::

ውይይት መንደርደሪያ ጥያቄዎች

1. ምን ያህል ህክምናው ከወሰዱ በኋላ አቋረጡ?
2. በጠቅላላ ያለው የቲቢ ህክምና አገልግሎት እንዴት ይገልፁታል?
3. ከቲቢ ህክምናው ጋር ተያይዞ ያገኙት አገልግሎት ነበር?
4. አስቸጋሪ ሁኔታ ሲገጥምዎት በአግባቡ ይረዱ ነበር? ከነበረ እንዴት ይገልፁታል?
5. በአጠቃላይ የቲቢ ህክምና ማዕከላዊነቱን እንዴት ይገልፁታል?
6. ማዕካላዊነቱን ለማሻሻል ምን ቢደረግ ይላሉ?
7. ባገኙት የቲቢ ህክምና እርካታዎ ምን ይመስላል?
8. የታካሚዎችን እርካታ ለመጨመር ምን ቢደረግ ይላሉ?
9. መመጨመር ወይም ማለት የምትፈልጉት ካለ?

ግዜዎን ሰጥተው ስለተሳተፉ አመሰግናለው።
